# Malaria in Pregnancy

**DOI:** 10.4084/MJHID.2013.010

**Published:** 2013-01-02

**Authors:** Ebako Ndip Takem, Umberto D’Alessandro

**Affiliations:** 1 Medical Research Council Unit, Fajara, The Gambia; 2 Institute of Tropical Medicine, Antwerp, Belgium

## Abstract

Pregnant women have a higher risk of malaria compared to non-pregnant women. This review provides an update on knowledge acquired since 2000 on *P. falciparum* and *P.vivax* infections in pregnancy. Maternal risk factors for malaria in pregnancy (MiP) include low maternal age, low parity, and low gestational age. The main effects of MIP include maternal anaemia, low birth weight (LBW), preterm delivery and increased infant and maternal mortality.

*P. falciparum* infected erythrocytes sequester in the placenta by expressing surface antigens, mainly variant surface antigen (VAR2CSA), that bind to specific receptors, mainly chondroitin sulphate A. In stable transmission settings, the higher malaria risk in primigravidae can be explained by the non-recognition of these surface antigens by the immune system. Recently, placental sequestration has been described also for *P.vivax* infections. The mechanism of preterm delivery and intrauterine growth retardation is not completely understood, but fever (preterm delivery), anaemia, and high cytokines levels have been implicated.

Clinical suspicion of MiP should be confirmed by parasitological diagnosis. The sensitivity of microscopy, with placenta histology as the gold standard, is 60% and 45% for peripheral and placental falciparum infections in African women, respectively. Compared to microscopy, RDTs have a lower sensitivity though when the quality of microscopy is low RDTs may be more reliable. Insecticide treated nets (ITN) and intermittent preventive treatment in pregnancy (IPTp) are recommended for the prevention of MiP in stable transmission settings. ITNs have been shown to reduce malaria infection and adverse pregnancy outcomes by 28–47%. Although resistance is a concern, SP has been shown to be equivalent to MQ and AQ for IPTp. For the treatment of uncomplicated malaria during the first trimester, quinine plus clindamycin for 7 days is the first line treatment and artesunate plus clindamycin for 7 days is indicated if this treatment fails; in the 2^nd^ and 3^rd^ trimester first line treatment is an artemisinin-based combination therapy (ACT) known to be effective in the region or artesunate and clindamycin for 7 days or quinine and clindamycin. For severe malaria, in the second and third trimester parenteral artesunate is preferred over quinine. In the first trimester, both artesunate and quinine (parenteral) may be considered as options. Nevertheless, treatment should not be delayed and should be started immediately with the most readily available drug.

## Introduction

### Epidemiology

Malaria in pregnancy (MiP) is a major public health problem in endemic countries. There is a wealth of evidence showing that the risk of malaria (both infection and clinical disease) is higher in pregnant than in non-pregnant women, possibly due to the immunological, hormonal changes or other factors occurring during pregnancy. Most of the available evidence is on *Plasmodium falciparum* and *P. vivax*, though for the latter, there is much less information than for *P. falciparum*, while little is known on *P. ovale* and *P. malariae*, the other two human malaria species. This review will focus on *P.falciparum* and *P. vivax*, with the objective of providing an update on the recently acquired knowledge (since the year 2000).

### Burden

Where transmission is stable and relatively high, mainly in sub-Saharan Africa, adults have acquired immunity against malaria, including pregnant women who, despite the immune tolerance occurring during pregnancy, are able to control but not clear malaria infections. Therefore, in this high risk group, asymptomatic infections are common while clinical malaria is relatively rare. A recent review of studies, carried out in sub-Saharan Africa between 2000 and 2011, reports that malaria prevalence in pregnant women attending antenatal clinics was 29.5% (95%CI: 22.4–36.5) in East and Southern Africa, and 35.1% (95%CI: 28.2–41.9) in West and Central Africa, while the prevalence of placenta malaria was 26.5% (95%CI: 16.7–36.4) in East and Southern Africa, and 38% (95%CI: 28.4–47.6) in West and Central Africa.^1^ More recently (studies published since 2008), the reported malaria prevalence (by microscopy unless specified otherwise) was lower, reflecting the recent decrease in malaria transmission observed in several African countries^2^–^11^ (Table 1). Most of the prevalence estimates were done by microscopy and they would probably be higher if more sensitive methods like PCR^12^ or placental histology^13^ were used. In addition, blood samples were collected at different times during pregnancy, increasing the difficulty of comparing different estimates.

In areas of low, unstable malaria transmission, mainly Asia-Pacific region and South America, pregnant women have a lower acquired immunity and malaria infections are more likely to evolve towards clinical disease. The number of pregnancies occurring in these areas has been estimated at 70.5 million in 2007.^14^ In the Asia-Pacific region, the median proportion of women with peripheral infection has been estimated at 15.3% and that of placenta malaria at 11%.^15^ For South and Central America, less data on the burden of malaria in pregnancy is available (Table 2). In Peru, the cumulative incidence of clinical malaria in pregnant women for the period January–August 2004 and 2005 was 43.1% as compared to 31.6% in non-pregnant women.^16^ This study also suggested that subclinical malaria infections may occur frequently among pregnant women in this region, despite the relatively low transmission, and that passive surveillance, i.e. data collection at health facilities, may underestimate the actual burden of MiP. In Colombia, the prevalence of malaria among parturient women attending the local hospital was 13% when determined by microscopy and 32% by PCR.^17^ In the same study, the prevalence of placenta malaria was 9% by microscopy and 26% by PCR, while 2% and 13% of cord blood samples were positive by microscopy and PCR, respectively.

### Risk Factors

Maternal factors associated with the risk of malaria in pregnancy include maternal age, parity and gestational age. It is well established that younger women (primigravidae and multigravidae), particularly adolescents, are at higher risk of malaria infection than older women,^18^–^20^ and this is independent of parity.^20^–^22^ Parity also affects the risk of malaria as primigravidae are at higher risk than multigravidae,^18^–^20^, ^23^–^24^ though less in low transmission settings,^15^ while in epidemic areas, the risk is not affected by parity.^25^ Most of the available data on malaria relate to the second and third trimesters.^12^, ^19^, ^26^–^27^ The peak of malaria prevalence seems to occur during the second trimester.^28^ Studies on malaria burden in the first trimester of pregnancy are scarce, but it is believed that the rates are similar to that of the second trimester. However, considering the difficulty of collecting this information (pregnant women start to attend the antenatal clinic after the first trimester), and of determining the gestational age with accuracy, it is unclear whether the risk starts to increase towards the end of the first trimester. Indeed, in Burkina Faso, malaria prevalence was higher during the first as compared to the second and third trimesters.^29^

### Effects of Malaria Infection

The effect of malaria infection during pregnancy will depend on the degree of acquired immunity, which in turn depends on the intensity of transmission.

#### Maternal effects

Where transmission is stable, such as in most of sub-Saharan Africa, most infections are asymptomatic but increase substantially the risk of anaemia.^19^,^26^,^30^–^31^ This occurs over a background of physiological anaemia of pregnancy due to increased blood volume. Both symptomatic and asymptomatic infections can cause anaemia. Severe anaemia is more often observed in stable transmission settings,^32^–^34^ and more in primigravidae than in multigravidae.^35^–^36^ Malaria infections in the first or second trimester of pregnancy increase the risk of anaemia,^24^,^30^ though one study reported an increased risk also for infections occurring in the third trimester.^30^ Preventing malaria infection by intermittent preventive treatment during pregnancy (IPTp) reduces the risk of anaemia.^27^,^37^–^38^

Where malaria transmission is unstable, malaria can cause maternal anaemia,^18^,^35^,^39^–^40^ more in primigravidae than in multigravidae and for falciparum infections more than for vivax infections.^18^,^35^ Nevertheless, severe anaemia is less common in these settings.^39^,^41^

In places where malaria transmission is stable, little is known on the importance of malaria infection as a cause of miscarriage. Where malaria transmission is unstable, malaria as a cause of miscarriage seems more common, as the majority of infections evolve towards a clinical attack with fever, which may by itself determine miscarriage. Indeed, non malarial fevers also independently increase the risk of miscarriage.^18^,^42^ Nevertheless, asymptomatic infections, i.e. slide confirmed malaria with no history of fever in the previous 48 hours and temperature <37.5 °C, was also associated with miscarriage.^7^

Maternal mortality associated to malaria is probably under-reported. Malaria was an important cause of maternal death in some studies,^43^–^45^ while in others it was not as frequent.^46^ The substantial reduction in maternal mortality observed in Thailand after the implementation of early detection and treatment of malaria suggests that malaria is an important contributor to maternal mortality.^47^ When not a direct cause of death (severe malaria),^47^ malaria in pregnancy is often reported as co-morbidity, e.g. with eclampsia, in conditions associated with maternal mortality.^44^,^48^

#### Perinatal effects

Malaria increases the risk of low birth weight (LBW),^19^,^23^,^30^,^49^–^51^ particularly in primigravidae, and this risk seems to be higher for infections in first or second trimester,^23^–^24^,^30^,^49^ though in one study this was true also for infections occurring late in pregnancy.^49^ In high malaria transmission settings, such an effect is due to intrauterine growth retardation (IUGR) rather than pre-term delivery, as most infections are asymptomatic. A meta-analysis of 32 cross-sectional data in Africa, showed malaria prevention in pregnancy is associated with 21% (95% CI= 14–27) reduction in LBW.^52^

In unstable transmission settings, preterm deliveries, still births and neonatal deaths have been associated with malaria.^18^*P.vivax* infections are also associated with LBW, and this effect appears to be similar in all pregnancies. In women with a single infection of *P.vivax* or *P.falciparum* detected and treated in the first trimester, no significant effect on gestation or birth weight was observed compared to women who also attended in the first trimester but who never had malaria detected in pregnancy.^42^

#### New born and infant effects

Fewer studies on malaria in pregnant women have evaluated infant outcomes. Congenital malaria can occur in the neonatal period and can contribute to infant morbidity and mortality.^53^ Placenta malaria, especially active infection, has been linked to neonatal and infant mortality.^53^ A recent study in The Gambia has showed that malaria infection during pregnancy influences infant’s growth, independently of LBW.^54^ It also increases the risk of infant’s death and perinatal mortality, by causing LBW.^39^,^53^,^55^ This is confirmed by the reduction neonatal mortality, up to 60%, observed after the implementation of preventive interventions targeted to pregnant women, e.g. intermittent preventive treatment.^56^–^57^ In primi- and secundigravidae, malaria prevention with IPTp or insecticide-treated bed nets was significantly associated with a 18% decreased risk of neonatal mortality.^52^

#### Later childhood, adolescence and adulthood effects

The long term effects of malaria in pregnancy have not been studied. However, malaria causes IUGR leading to LBW, which may be related to diseases occurring during adulthood, including some cancers and the metabolic syndrome.^58^

## Pathophysiology

Pregnant women are at higher risk of contracting malaria than non-pregnant women. This increased susceptibility can be explained by the immunological changes induced by pregnancy, by hormonal factors,^59^ and by the higher attractiveness of pregnant women to mosquitoes.^60^–^61^ In addition, *P. falciparum* -infected erythrocytes in pregnant women bind to specific receptors, i.e. chondroitin sulphate A (CSA), and sequester in the placenta.^62^–^63^ They rarely bind to the other two commonly described receptors in non-pregnant individuals, i.e. CD36 and the intracellular adhesion molecule (ICAM-1). In pregnancy, the parasite antigens expressed on infected erythrocytes are collectively known as variant surface antigen-pregnancy associated malaria (VSA_PAM_). They are different from those expressed in non-pregnant individuals and in stable transmission settings are not recognised by the immune system, explaining the higher risk in primigravidae.^64^ The binding of the variant surface antigen (VAR2CSA) with chondroitin sulphate A has been implicated in the pathology of falciparum malaria in pregnancy.^65^–^68^ The VAR2CSA belongs to the family of the erythrocyte membrane protein (PfEMP1), is encoded by the var2csa gene and its expression has been described in pregnant women with falciparum malaria.^69^ Levels of anti-VAR2CSA specific IgGs increase with parity, cannot be found in men and are associated with a favourable pregnancy outcome 64–66 so that the malaria risk decreases with increasing parity. Besides the antibody responses to VSA_PAM_, cytokine responses such as Th1, Th2, interleukins, TNF and regulators, IFN gamma,^70^–^72^ and monocytes^73^ have been observed in pregnant women with malaria. Rosetting, a phenomenon consisting of parasite-free erythrocytes surrounding parasite-infected erythrocytes and commonly observed in non-pregnant individuals, has been implicated in the pathogenesis of severe malaria^74^–^75^ but is uncommon in pregnant women with falciparum malaria.^76^

The sequestration of *P. vivax* in the placenta, though until recently thought not to occur, has been described,^77^–^78^ with the involvement of ICAM-1 and CSA as receptors.

The effects of hormonal changes on pregnancy associated malaria have been described in few studies and are subject to debate. Increased cortisol levels have been associated with increased risk of malaria in pregnant women.^79^

The increased attractiveness of pregnant women to mosquitoes may be explained by physiological and behavioural changes occurring during pregnancy. Physiological changes include increased exhaled breath and increased abdominal temperature that may render pregnant women more easily detectable by mosquitoes. Behavioural changes are represented by the fact that pregnant women urinate twice as frequently as non-pregnant women, resulting in an increased exposure to mosquito bites at night because they have to leave the protection of their bed nets.^60^–^61^

Malaria-associated placental changes have been described for stable^72^,^80^ and unstable transmission settings.^73^,^81^ They include presence of parasites, inflammatory changes and hemozoin (pigment) deposition. Placental changes have been characterised into four levels, i.e. acute (parasites present, malaria pigment absent), chronic (parasites and malaria pigment present), past infection (no parasite but pigment present) and no infection (both parasites and malaria pigment absent).^82^ Recently, a 2-parameter grading system, distinguishing between inflammation and pigment deposition, has been proposed as it correlates with pregnancy outcomes, in both a stable transmission setting in Tanzania, and an unstable setting in Thailand.^73^

It is unclear what the mechanism at the basis of malaria-related preterm delivery is, though fever, anaemia, and high levels of TNF alpha or interleukin 10 have been identified as important risk factors.^18^,^83^–^84^

LBW due to IUGR is associated with maternal anaemia,^83^,^85^ and elevated levels of cytokines.^70^ Although the exact mechanism has not been elucidated, it appears to be due to chronic infections that cause reduced foetal circulation and placental insufficiency.^86^ Placental endocrine changes related to falciparum infection have been suggested as another possible mechanism leading to IUGR.^87^

*P.vivax* is different from *P. falciparum* as it infects immature erythrocytes (reticulocytes), limiting the parasite densities. In addition, it can relapse during pregnancy due to the activation of liver hypnozoites. Vivax parasites do not frequently express variant surface antigens, at the basis of placenta sequestration, so that this does not occur frequently.^81^ Therefore, *P. vivax* probably affects birth weight, and increases the risk of miscarriage and preterm birth through a systemic rather than a local effect. Nevertheless, the mechanisms at the basis of these observations are not completely understood.

## Clinical Presentation

### Diagnosis

The diagnosis of malaria in pregnancy is essential to prevent its deleterious effects to the mother and the foetus. Unfortunately, the clinical signs of malaria in pregnant women are usually non specific, and where transmission is stable, most infections are asymptomatic. Therefore, suspected malaria cases should be confirmed by parasitological diagnosis,^88^ usually by microscopy and/or rapid diagnostic tests. Nevertheless, other methods such as PCR and placental histology can be also used, though the latter can be done only after delivery so that it cannot be used for the management of infections occurring during pregnancy.

Microscopy is one of the most widely used methods for diagnosing malaria, including during pregnancy. It has some advantages such as the possibility of determining the parasite density and species. However, its major disadvantage, besides the need of a regular power supply, is its sensitivity, which cannot go below 10–15 parasites per μl. Therefore, a substantial proportion of infected pregnant women would not be detected because of extremely low parasite densities or of parasites sequestered in the placenta, though both conditions have deleterious effects on the mother’s and her offspring’s health.

Several studies have investigated the use of microscopy for the diagnosis of MiP in stable malaria transmission settings in Africa.^89^–^91^ When taking placenta histology as the reference test, the sensitivity of peripheral blood microscopy for _*P. falciparum* infections (4 studies) was 60% (95% CI=50–69) and that of placental microscopy 45% (95% CI=34–56).^13^

In settings with unstable malaria transmission, there are few studies on the sensitivity of microscopy on peripheral blood collected during pregnancy.^13^

Rapid diagnostic tests (RDT), detecting circulating malaria antigens, can also be used. Generally, the sensitivity of RDTs for the diagnosis of malaria in pregnancy is lower than that of microscopy. However, the time needed for the diagnosis is shorter than for microscopy and the training required for their use is minimal. Although RDT can detect malaria antigens, they cannot estimate the parasite density. The sensitivity of RDT on peripheral blood using peripheral microscopy as a reference test is estimated at 81% (95% CI= 55–95), and the sensitivity of RDT on placental blood was 81% (95% CI= 62–92) using placental microscopy as the reference.^13^

PCR, which detects parasite DNA, can also be used for the diagnosis of malaria infection but is not readily available in health facilities. In stable transmission settings, the sensitivity of PCR was >80% when using microscopy as the reference.^13^ PCR sensitivity has not been estimated against placental histology as reference test.

### Severe malaria

Severe malaria in pregnancy is more common in unstable transmission settings because of the lower immunity pregnant women have. Generally, women in the second and third trimesters of pregnancy are at a higher risk of developing severe malaria compared to non-pregnant adults. In low transmission settings, severe malaria in pregnancy is usually associated with pulmonary oedema, hypoglycaemia and severe anaemia. Mortality in pregnant women with severe malaria and treated with either artesunate and quinine varied between 9% and 12%.^92^

## Prevention and Treatment

### Prevention

The most widely used interventions to prevent malaria in pregnancy are insecticide-treated bed nets (ITN), including Long-Lasting Insecticidal Nets (LLINs), and intermittent preventive treatment in pregnancy (IPTp).

While ITNs have shown a substantial reduction in malaria morbidity and mortality in children,^93^–^94^,^95^,^96^ in pregnant women, it has been associated with a decrease in maternal parasitaemia (38%), anaemia (41%) and LBW (28%),^97^ and 47% reduction in maternal anaemia.^98^ In one study, there was no evidence of a reduction in anaemia and parasitaemia.^99^

IPTp is the administration of therapeutic doses of an antimalarial, currently sulfadoxine-pyrimethamine (SP), at least twice during pregnancy, in the second and third trimester, irrespective of the presence of a malaria infection. The WHO recommends its use and many sub-Saharan African countries have included it in their malaria control program. In stable transmission settings, many trials have shown that SP given as IPTp is efficacious in preventing the adverse consequences of malaria during pregnancy (Table 3).^100^–^104^ However, SP resistance represents a major threat. A study in Benin has showed that, despite the presence of molecular markers of resistance, SP remained efficacious.^105^ This has been confirmed by a review reporting that IPTp with SP is effective up to a certain level of SP resistance.^106^ Nevertheless, finding an alternative to SP for IPTp is important. Adding amodiaquine to SP was efficacious but not better than SP alone.^107^ Mefloquine (MQ), thanks to its long elimination half-life, could be a good alternative to SP as it would provide a long post-treatment prophylactic period. Indeed, a trial in Benin showed that for IPTp MQ was as good as SP in preventing LBW. MQ was more efficacious than SP in preventing placental malaria, clinical malaria and maternal anaemia at delivery. However, MQ was less well tolerated than SP, potentially compromising its large scale use as IPTp.^108^–^109^

There is no evidence that one of the methods is better than the other^110^ and the combined use appears to be better than individual use.

A different approach is systematic screening for malaria infections at regular intervals and treatment of the positive women, which may be more appropriate in settings where malaria transmission is low and the risk of infection between antenatal visits is also low. It has already be shown to have similar protective efficacy than IPTp but additional trials for a more thorough evaluation of this intervention are probably needed.^26^ Due to drug resistant malaria, it has been the only form of malaria control on the Thai-Burmese border for more than 20 years, impacting significantly on maternal mortality rates.^47^

In future, vaccines specifically designed to prevent MiP may become available; VAR2CSA, in the early stages of development, seems the most promising candidate.^111^–^116^ However, there are still several uncertainties, including the number of antigenic variants to be combined for an optimal response, the timing of the vaccine, e.g. during pregnancy or at puberty, whether only first pregnancies should be targeted, and the length of follow up for children born to vaccinated mothers.^111^–^112^,^117^

### Treatment

It is recommended that pregnant women with malaria are treated after parasitological confirmation of the diagnosis, reducing the unnecessary exposure to antimalarials of both the mother and the foetus.

#### First trimester

Clinical trials on the safety and efficacy of antimalarials in pregnancy usually exclude women in the first trimester of pregnancy so that the evidence is based on observational studies (Table 4). Artemisinin derivatives were relatively safe (n=1937) in the first trimester of pregnancy^42^,^118^–^119^ and the cumulative failure rate reported in only one study was 6.6% across all trimesters (n=461).^118^ No major adverse event was observed in 377 women with known pregnancy outcome and exposed to artemisinins in the first trimester.^42^,^119^–^121^ However, only 1 study^120^ out of 4, was a randomised controlled trial though the treatment was given during a mass campaign and the exposure was thus inadvertent; the birth weight of newborns delivered by women exposed to artesunate during the first trimester was similar to that of the other pregnant women. According to recommendations,^88^ chloroquine, quinine, clindamycin and proguanil can be considered safe in the first trimester.

In case of uncomplicated malaria in the first trimester, a combination of quinine + clindamycin for 7 days is recommended.

In case of severe malaria, parenteral antimalarials are recommended.^88^ In the first trimester, the risk of hypoglycaemia is lower and the uncertainties on the safety of the artemisinins derivatives are greater. Nevertheless, considering that treatment should not be delayed and that artesunate reduces the risk of death, both artesunate and quinine (parenteral) may be considered as options. Treatment should be started immediately with the most readily available drug.^90^

#### Second and third trimesters

There is more experience on the use of artemisinin derivatives in the second and third trimesters of pregnancy. Evidence is available from both trials^122^–^127^ and observational studies^128^–^131^ involving pregnant women (Table 4). Data available indicate that ACTs are relatively safe for the foetus when taken after the first trimester of pregnancy. A recent review of treatment studies carried out in pregnant women from 1998–2009, reported a parasitological failure >5% in 3 out of 11 trials.^132^ In the second trimester, ACTs that are known to be effective in the area, or 7 days artesunate+ clindamycin, or 7 days quinine+ clindamycin are recommended for uncomplicated malaria.^88^ In case of severe malaria, parenteral artesunate is preferable because it saves the life of the mother. Several studies have shown that the kinetics of artemisinins derivatives, most specifically of the active metabolite dihydroartemisinin, is modified during pregnancy.^133^–^134^

Amodiaquine (AQ) has been shown to be efficacious in pregnant women with falciparum malaria in Ghana and Tanzania.^122^,^125^ Day 28 parasitological failure rates were 3% for AQ monotherapy,^122^ 0–1% for the combination AQ+SP,^122^,^125^ and 4.5% for the combination AS+AQ.^125^ It was relatively safe and well tolerated and associated with some minor side effects (nausea, weakness, dizziness). Blood dyscrasias were not a problem associated with its use. A pharmacokinetics study on AQ for treatment of *P.vivax* in pregnancy conducted in Thailand indicates the doses are similar to that of non-pregnant adults.^131^,^135^

There are fewer reports on the efficacy and safety of mefloquine (MQ) for MiP. High cure rates have been reported in Thailand, for the combination of MQ+AS (cure rate of 98.2% at day 63).^126^ One study reported minor side effects.^109^ However, there are concerns about still births and neuropsychiatric disorders. There are currently some ongoing clinical studies which will provide useful data on the safety, efficacy and pharmacokinetics of MQ in pregnant women (Table 5). The combination AS+ MQ is being evaluated in studies in Africa and Asia (NCT00852423, NCT00701961, NCT01054248, CTRI/2009/091/001055TEMP, NCT01054248).

In Uganda, in an area of relatively high transmission and hence with pregnant women having some acquired immunity, artemether-Lumefantrine (AL) was efficacious, with cure rates >95%.^136^–^137^ However, in Thailand the cure rate at day 42 was only 82%,^138^ possibly due to the low day 7 lumefantrine concentrations. AL was safe and well tolerated.^136^–^138^ As for other antimalarial treatments, pharmacokinetics may be altered during pregnancy, with plasma concentrations lower than expected.^129^,^139^ AL is currently being evaluated in Thailand and in four sites in sub-Saharan Africa (NCT01054248, NCT00852423).

Dihydroartemisinin piperaquine (DHAPQ) was highly effective in women with multiple recrudescent infections on the Thai-Burmese border.^140^ DHAPQ is used in the Western Pacific for malaria in pregnant women.^141^ DHA-PQ is currently being evaluated in 3 studies in Africa and Asia (NCT00852423, NCT01054248, NCT01231113). Cure rates and PK are reassuring.

In Thailand, atovaquone-proguanil in combination with artesunate (AAP) was associated with high cure rates (>95%) and was relatively safe,^123^,^142^ though the sample size was small. In Thailand, plasma concentrations of AAP were lower in pregnant than in non pregnant women.^143^

### Conclusions

This review shows that although the deleterious effects of MiP to both the mother and the child are well documented, the mechanisms involved are still relatively unknown, particularly where transmission is low and unstable. The diagnosis of MiP is challenging, as peripheral microscopy will miss a large proportion of infected women with parasites sequestered in the placenta. MiP can be prevented by currently available control methods, i.e. ITNs and IPTp, but the challenge is attaining a high coverage, particularly for women with the highest risk such as adolescent primigravidae. It is still unclear what would be the alternative to SP for the IPTp.

The burden of *P. vivax* MiP, which is substantial in the Asia-Pacific region and in South America has been relatively neglected. It is generally believed that vivax infections are milder than falciparum ones, but this is based on few studies. There is also the need of having more sensitive diagnostic methods for vivax infections, as it would help improving early diagnosis and appropriate management. Finally, information of the safety and efficacy of antimalarials during pregnancy is growing, though this is true mainly for the second and third trimester. For the first trimester, treatment options are still extremely limited and evidence is mainly based on pharmacovigilance data on accidental exposures.

## Figures and Tables

**Table 1:** Burden of malaria in pregnancy in sub-Saharan Africa

Country	Year	N	Parasite prevalence		Trimester	Diagnosis	Species*
			peripheral	placenta			
Burkina Faso [23]	2006–2008	1034	39.2^a^		1–3	M	F
Nigeria [19]		396	18 b		2,3	M	F
Benin [30]	2008–2010	982		11.5^i^	1–3	M, RDT, H	
Ghana [90]	2000	596		32, 38, 56 c			F
Ghana [27]	2009	363	28.40		3	M	
Burkina [49]	1987–1988	1190	5–11^d^	8.7			F >90%
Kenya [36]	1996–1997	912	10–24	30–64^e^	2,3	M, H	
Tanzania [144]	2003–2004	413		8%	1,2		
Cameroon [22]	1996–1998	278	22.6, f 76.1	26.8, 52.9		M, PCR	F, others
Malawi [24]	2002–2003	1869	20.1			M	
The Gambia[54]	2002–2005	783		9.5		H	
Cameroon [71]	2002	175		25.4			
Kenya [50]	2003	85		44–81	1–3	H	
Nigeria[80]	2002	304		33.2		H	
Senegal [72]		692		10^h^		RDT, M	
Cameroon [34]	1998–2000	1143		44.7		M	
Cameroon [51]	1999–2001	770	32.8	33.7		M	
Malawi[12]	2003–2006	475	2.30			M	
Angola[145]	2008	679	10.9		1–3	M	F
Burkina [29]	2003	295, 288	11.9, 32.2^j^		1–3	M	F
Gabon [31]	1995–1996	311	57			M	F

* F=*Plasmodium falciparum*; M: Microscopy, H: Histology;

a incidence in per thousand women months;

b peripheral and placental;

c microscopy, RDT and PCR respectively;

d according to trimester;

e based on histology (primigravidae-multigravidae);

f microscopy, PCR;

g peripheral and placental;

h RDT;

i 80/696;

j dry season, transmission season

**Table 2:** Malaria burden in pregnancy in Asia-Pacific and South America

			Parasite prevalence				
Country	Year	N	peripheral	placenta	trimester	diagnosis	species
Thailand [42]	1986–2010	17613	5*		1	M	F, V
India[20]	2006–2007	2386 a718	1.8	2.4	2,3	M, RDT	F, V, mixed
Peru[16]	2004–2005	1645–1652^b^	8.1–6.6			M	F, V
Venezuela[40]	200–2002	12					V
Thailand [81]	1995–2002	204	96.0^f^	6.9%	1,2,3	M	F, V, mixed
Brazil[146]	1997	195	67.7 c, 29.7				F, V, mixed
Thailand[39]	1993–1996	1459	37		1,2,3	M	F, V, mixed
Indonesia[141]	2004–2010	4478	19		2,3	M	
Colombia[17]		84		13^d^		M, PCR, RDT	
Ecuador[21]	2001		56.3				F
Peru[73]	2004	193	1 e 6.6	0.53 5.17		M, PCR	F, V

* F=*Plasmodium falciparum*; V= *Plasmodium vivax*; M: Microscopy, H: Histology;

a Antenatal clinics, delivery units;

c vivax, falciparum-pregnant women were symptomatic (fever);

d microscopy;

e microscopy, PCR;

* estimated from table;

f estimated from data presented in paper (175/402)

**Table 3:** Trials on Intermittent Preventive Treatment in pregnancy

Country	Year	Trial arms	N	Findings
Uganda[110]	2004–2007	SP vs SP+ITN vs ITN+placebo	5775	No differences between treatment arms
Mali[100]	2006–2008	SP 3 vs 2 doses	814	SP3 vs SP2: 50% reduction in placental parasitaemia, LBW, pre-term births
Ghana[107]	2004–2007	SP, AQ, SP+AQ	3643	No difference peripheral parasitaemia, adverse events more frequent with AQ
Benin[109]	2005–2008	MQ, SP	1601	No difference in LBW, MQ more efficacious than SP in preventing malaria, MQ had more adverse events
Burkina Faso [37] **	2004–2006	SP	1441	SP2 vs SP0^c^ At delivery, 96% reduction placental infection, increase PCV, reduction LBW in primigravidae
Benin[108]	2005–2006	CQ, SP	1699	SP vs CQ decreased LBW by 50%, placental infection by 80%
Mozambique[101]	2003–2005	SP, PB^a^	1030	No reduction of LBW, anaemia at delivery and placenta malaria; 40% reduction incidence of clinical malaria
Mozambique*[57]	2003–2005	SP, PB^a^	1030	PE 61.3% neonatal mortality
Ghana[26]	2007–2008	IPTp-SP, IST	3333	No difference between study arms but increase in Hb after intervention
Nigeria[102]	2003–2005	SP, CQ	352	PE against anaemia: 49.5 SP vs CQ
Nigeria[103]	2002	SP, CQ-P	500	SP better than CQ-P
Mozambique[104]	2001–2002	SP vs??	600	Parasite prevalence SP 6.3% vs 13.6%, 2.4 vs 13.3, high loss to follow-up

a PB=placebo, SP=sulfadoxine-pyrimethamine, AQ=amodiaquine, CQ=chloroquine, P=pyrimethamine;

b 12–28 weeks;

c SP2=2 doses of SP, SP0=no dose of SP;

* maternal and birth outcomes up to 8 weeks have been reported in [101];

** community trial;

PE=parasitological efficacy; IST: Intermittent Screening and Treatment

**Table 4:** Treatment trials and clinical studies on malaria in pregnancy

Country	Year	Antimalarial	N	Trimester	Findings
Uganda[136]	2006–2009	Q, AL	304	2,3	day 42 CR: AL 99.7% Q 97.6; Q group more adverse events
Ghana[122]	2003–2004	CQ, AQ, SP, AQ+SP	900	2,3	day 28 PF: CQ 14%, SP 11%, AQ 3%, AQ+ SP 0%.
Tanzania[125]	2004–2006	SP, CD, AQ+SP, AS+AQ	272	2,3	day 28 PF: CD 18%, AQ+SP 1%, AS+AQ 4.5%.
Uganda[137]	2006	AL, CD	114	2,3	Day 28 CR: AL 100% CD 100%.
Thailand[138]	2004–2006	AL, AS7	252	2,3	Day 42 or delivery CR: AS7 89.2%, AL 82%.
Thailand[126]	1995–1997	Q, AS+MQ	108	2,3	Day 63 CR: AS+MQ 98.2%, Q 67%,
Thailand[123]	2001–2003	AAP, Q*	81	2,3	Day 63 CR: AAP 94.9%, Q 63.4%
Thailand[142]	1999–2001	AAP	27		Day 42 CR: 96%
Malawi[127]	2003–2004	SP, SP+AZ, SP+AS	141	2,3	PF: SP-AS^a^ 14.3%, 11.4%, 44.8 %, Recrudescence less frequent in SP-AS vs SP (HR 0.25)
Thailand[124]	1997–2000	QC7, AS7	129	2,3	both had 100% day 42 CR
Uganda^c^[128]	2008	A, DHA	21	2,3	PK concentrations A and DHA lower than non pregnant adults
Thailand[135]		AQ	27		AQ reduced recurrent infections from 22.2 to 7.4% day 35 for *P.vivax*, PK PD no adjustments of dose required
PNG [147]	2006	CQ for IPTp	30	2,3	Reduced plasma concentrations CQ and metabolite
Mali, Mozambique, Sudan, Zambia[148]	-	SP for IPTp	97	2,3	PK Inconsistent changes in concentrations of S and P
Thailand b [129]	2004–2006	L in AL	103		PK: 40% low capillary concentrations
Thailand[133]	-	DHA&PQ	24		PK: Reduced exposure DHA, unaltered exposure PQ
Thailand[131]	2007–2008	AQ	24	2, 3	PK: Safe, similar pharmacokinetic properties with non pregnant
Thailand[139]	2004	AL	13	2,3	PK: Reduced plasma concentrations of both A and L
Thailand[134]	2000–2001	DHA	24	2,3	PK: DHA lower
Thailand[118]	1992–2000	AS, A, sometimes in combination with MQ, co-A, AQ-PG	461	1	cumulative artemisinin PF: 6.6%; retreatment: 21.7
Thailand[149]	1995–2000	Q-PF, CQ-PV	300	1	safe but more tinnitus and maternal anaemia for Q
Thailand[42] ‡	1986–2010	Q, CQ-PV, AS, MQ	17613	1	miscarriage risk asymptomatic malaria OR=2.7, symptomatic malaria OR=3.99; no significant effect of drug on miscarriage or malformation rates
Zambia[119]	2004–2008	AL, SP	1001	1	perinatal mortality OR AL vs SP 0.84(0.45–1.53), no difference in maternal mortality, still birth, LBW; increase abortion rate AL

AS=artesunate, A=artemether, AQ=amodiaquine, AZ=Azithromycine, C=clindamycin, CD=chlorproguanil-dapsone, DHA=dihydroartemisinin, PQ=piperaquine, CQ=chloroquine, L=lumefantrine, MQ=mefloquine, Q=quinine, SP=sulfadoxine-pyrimethamine; PF=parasitological failure; CR=clearance/cure rate; PE=parasitological efficacy; PK=pharmacokinetics; PNG=Papua New Guinea;

* supervised quinine for 7 days;

a peripheral microscopy, placental microscopy and placental histology;

‡ part of data in McGready 2001 [118], Mc Gready 2002 [149];

b data from McGready 2008[138];

c main trial Piola 2010[136]

**Table 5:** Registered ongoing trials on malaria treatment in pregnant women

Study	Country	Registration ID	intervention
Effective and safe treatment for malaria in pregnancy in India: a randomised controlled trial	India	CTRI/2009/091/001055TEMP	AS+SP, AS+MQ
Randomized trial of 3 artemisinin combination therapy for malaria in pregnancy (DMA)	Thailand	NCT01054248	AS+MQ, AL, DHA+PQ
Safe and efficacious artemisinin-based combination treatments for African pregnant women with malaria (PREGACT)	Burkina Faso, Ghana, Malawi, Zambia	NCT00852423	DHA-PQ, AS+MQ, AS+AQ, AL
Pharmacokinetics of mefloquine-artesunate in Plasmodium falciparum malaria infection in pregnancy	Burkina Faso	NCT00701961	MQ-AS (pregnant vs. non- pregnant)
ACT in pregnant women	Nigeria	PACTR2010020001862624	Experimental group: AS/AQ, control group AL
Efficacy, safety and tolerability of dihydroartemisinin- piperaquine for treatment of uncomplicated malaria in pregnancy in Ghana	Ghana	NCT01231113	Drug: AS-AQ Drug: DHA-PQ fixed-dose combination
